# T‐cell modulation by cyclophosphamide for tumour therapy

**DOI:** 10.1111/imm.12913

**Published:** 2018-03-09

**Authors:** Ellyn Hughes, Martin Scurr, Emma Campbell, Emma Jones, Andrew Godkin, Awen Gallimore

**Affiliations:** ^1^ Division of Infection & Immunity School of Medicine Cardiff University Cardiff UK; ^2^ Present address: Faculty of Medicine Nursing and Health Sciences School of Biomedical Sciences Monash University Melbourne Australia

**Keywords:** cancer, cyclophosphamide, regulatory T cells, T cells

## Abstract

The power of T cells for cancer treatment has been demonstrated by the success of co‐inhibitory receptor blockade and adoptive T‐cell immunotherapies. These treatments are highly successful for certain cancers, but are often personalized, expensive and associated with harmful side effects. Other T‐cell‐modulating drugs may provide additional means of improving immune responses to tumours without these disadvantages. Conventional chemotherapeutic drugs are traditionally used to target cancers directly; however, it is clear that some also have significant immune‐modulating effects that can be harnessed to target tumours. Cyclophosphamide is one such drug; used at lower doses than in mainstream chemotherapy, it can perturb immune homeostasis, tipping the balance towards generation of anti‐tumour T‐cell responses and control of cancer growth. This review discusses its growing reputation as an immune‐modulator whose multiple effects synergize with the microbiota to tip the balance towards tumour immunity offering widespread benefits as a safe, and relatively inexpensive component of cancer immunotherapy.

## Introduction

Mustard gas, a poison used during World War I, damaged eyes, skin and lungs causing great suffering and death to thousands of soldiers. Autopsies performed on some of the soldiers who died in this way, revealed shrunken lymph nodes and depleted bone marrow while the medical records of others revealed low immune cell numbers in their blood (reviewed in ref. 1). Louis Goodman and Alfred Gilman, both doctors at Yale University, hypothesizing that mustard gas destroyed immune cells, tested its ability to treat a patient with advanced lymphoma.^1^ Far from the horror of the trenches, clear benefits of this toxic substance were observed, signifying the advent of chemotherapy.^1^

With these discoveries, mustard gas served as a starting point to produce similar but less toxic and more effective anti‐cancer agents. One of these was cyclophosphamide (CY), becoming one of the earliest anti‐cancer drugs first used in the late 1940s. It is now known that CY is metabolized in hepatocytes by P450 oxidases to produce its active forms namely 4‐hydroxycyclophosphamide and its tautomer aldophosphamide.^2^, ^3^ Once inside a cell, these undergo spontaneous degradation into acrolein and the alkylating agent, phosphoramide mustard, resulting in DNA cross‐linking DNA and apoptosis. Currently, CY is used as a chemotherapy agent for a range of cancers and as an immunosuppressive drug to treat autoimmune conditions refractory to standard therapies; novel uses of CY as a cancer immunotherapeutic agent are also being explored.

## CY and tumour immunity

Robert North and colleagues demonstrated the impact of CY on tumour immunity in the 1980s. Interpretation of these experiments was linked to the discovery of a ‘suppressor’ T‐cell population capable of promoting tumour growth through dampening down the anti‐tumour immune response. In these experiments, T cells induced by immunizing mice with fibrosarcoma cells admixed with killed *Corynebacterium parvum*, caused tumour regression when adoptively transferred into tumour‐bearing T‐cell‐deficient mice, but not when adoptively transferred with T cells recovered from tumour‐bearing mice, consistent with the hypothesis that tumours induce suppressor T‐cell activity.^4^ Administration of 100 mg/kg CY was subsequently shown to abrogate the suppressive effect. Importantly, in the absence of T cells, CY alone had little or no effect on tumour growth, indicating immune‐modulation by CY, consistent with its ability to ablate suppressor T‐cell activity.^4^, ^5^ Further evidence for an important role for CY in immune‐modulation was obtained when the same group investigated the effects of CY on anti‐tumour immunity using a CY‐resistant lymphoma cell line (L5178Y). Regression of the lymphomas was observed if CY was co‐administered with donor lymphocytes obtained from tumour‐sensitized animals; the minimum CY treatment required was 70 mg/kg. These results indicate that CY can impart an anti‐tumour effect without a direct impact on the tumour itself.^5^ Many recent studies using CY, described in detail below, support the interpretation of these early reports.

## CY and regulatory T cells

Many more studies have since confirmed that ‘suppressor’ T cells, subsequently defined as CD4^+^ CD25^hi^ Foxp3^+^ regulatory T (Treg) cells are often enriched within tumour sites where they may inhibit the activity of anti‐tumour T cells and promote tumour progression.^6^ Several suppressive mechanisms used by Treg cells have been reported, all of which may contribute to the immunosuppressive tumour microenvironment, allowing the cancer to progress (reviewed in ref. 7). It follows that strategies designed to inhibit Treg cell activity should facilitate productive anti‐tumour immune responses. It is still the case however, that specifically depleting Treg cells or blocking their ability to function/accumulate is challenging because of a paucity of unique, targetable markers (reviewed in ref. 7). The possibility of using CY to selectively manipulate these cells as part of a cancer immunotherapy is therefore attractive.

Studies exploring the impact of CY on tumours and/or the immune system have revealed that in humans, although high doses of CY are needed for effective chemotherapy (400–1000 mg/m^2^) and for inducing systemic immunosuppression, immune‐modulation requires much lower doses (< 300 mg/m^2^ or ~8 mg/kg). In the case of rodent models, different laboratories using human equivalent doses of CY (~ 100 mg/kg) consistently report a decline in Treg cells following treatment of mice and rats with CY, probably because of enhanced cell death and decreased homeostatic proliferation.^8^, ^9^, ^10^, ^11^, ^12^, ^13^ Hence, alterations in Treg cell proportions probably reflect both alterations in cell number and changes in the balance of different lymphocyte sub‐populations induced because of CY. The extent and kinetics of alterations in lymphocyte populations following exposure to CY appear to depend on dose (higher CY doses are more likely to induce lymphopenia) and other factors influencing immune activation. Detailed studies examining the effects of CY on Treg cell function have revealed that Treg cells recovered from CY‐treated animals are more apoptosis‐prone and less suppressive than those from untreated mice; these effects are not long‐lasting because Treg cells appear to regain normal function within 10 days after administration of CY.^8^, ^12^, ^14^

Gene expression analyses conducted on blood, bone marrow and spleen at various time‐points after CY administration have proven informative in shedding light on why so many immunological changes are induced. Moschella *et al*. revealed that exposure to CY results in up‐regulation of gene signatures for cell death, DNA repair, pro‐inflammatory cytokines/chemokines, and T helper type 1 (Th1)/Th17 responses, among others.^15^ These changes were most pronounced early after administration of CY (1 day later) implying that immune activation is triggered as a direct consequence of the cytotoxic effects of CY. There is also evidence that transient depletion of bone marrow cells by CY induces a rebound myelopoiesis, thereby further perturbing immune homeostasis, potentially affecting myeloid cells as well as lymphocytes. Indeed, CY administration has been reported to increase the frequency of myeloid‐derived suppressor cells, which may serve to limit its immune‐potentiating effects.^16^, ^17^, ^18^

## Why does CY target Treg cells?

It has been suggested that low‐dose CY selectively targets Treg cells due to the relatively low‐levels of ATP present in these cells compared with other lymphocytes.^19^ This is due in part to inhibition of microRNA‐142‐3p by Foxp3, the consequence of which is increased synthesis of adenyl cyclase 9, which drives conversion of ATP into cAMP. Reduced ATP is significant, as one detoxification method used by cells to remove active phosphoramide mustard involves the conjugation of phosphoramide mustard to glutathione, which is itself synthesized via two ATP‐dependent reactions.^19^, ^20^, ^21^ Hence, as a consequence of reduced ATP, Treg cells harbour relatively low levels of glutathione and as a result fail to efficiently detoxify CY, whereas addition of glutathione to Treg cells attenuates their sensitivity.^19^ Moreover, the importance of microRNA‐142‐3p has been validated in experiments where Treg cells transfected with microRNA‐142‐3P exhibited an increase in levels of intracellular ATP and resistance to CY. Conversely, the introduction of a microRNA‐142‐3P inhibitor to conventional T cells led to a decrease in ATP levels and enhanced sensitivity to CY‐mediated killing *in vitro*. The ectonucleotidases CD39 and CD73, expressed on the surface of Treg cells, may also play a role since catabolism of extracellular ATP to adenosine may lead to a decrease in cytosolic ATP by inducing its release. Antagonists of CD39 increase ATP levels in human and mouse Treg cells, also resulting in resistance to CY.^22^, ^23^, ^24^

## Cyclophosphamide for cancer immunotherapy – pre‐clinical mouse models

Following the studies of Robert North in the 1970s and 1980s, the ability of CY to exert indirect anti‐tumour effects through its immune‐modulating activity have been demonstrated.^9^, ^25^ Motoyoshi *et al*. compared the effect of low (20 mg/kg) or high (200 mg/kg) doses of CY in immunocompetent and nude mice bearing hepatocellular carcinomas (MH129). An anti‐tumour effect was seen at both CY doses in the immunocompetent mice, but was only observed at a high dose in the immunodeficient mice, indicating direct tumour cytotoxicity at a high dose of CY but an indirect immune‐modulating effect at the lower dose.^25^ The same group also showed that adoptive transfer of purified Treg cells to CY‐treated tumour‐bearing mice negates the ability of CY to promote tumour immunity.

Through these studies, an emerging consensus is that CY results in a reduction in the proportion (and possibly function) of Treg cells, with a concomitant increase within the CD4^+^ Foxp3^−^ and CD8^+^ effector T‐cell compartments. Several reports indicate that the net effect of this immune perturbation is to drive anti‐tumour immune responses, as evidenced by increased numbers of tumour‐infiltrating effector T cells and/or control of tumour progression.^9^, ^10^, ^11^, ^13^, ^26^ There are also studies indicating that CY treatment results in an increase in production of type I interferon, driving maturation of dendritic cells.^27^ This effect together with tumour cell death and consequent antigen release may be crucial for driving anti‐tumour T‐cell responses.^28^ Recent evidence using mouse models indicates that enhancing tumour immunogenicity through chemotherapy (including CY) ‐induced cell death is highly effective at improving responses to checkpoint blockade.^29^

Although administration of CY alone can significantly impair tumour growth in mice,^30^, ^31^ there is also evidence from mouse models that the ability of CY to promote anti‐tumour immunity is markedly improved by combining the drug with other chemotherapeutic^32^, ^33^ or immunotherapeutic agents. In the case of the latter, investigators have demonstrated increased immune responses and better control of tumour growth after combining low‐dose CY with recombinant virus vaccines,^34^ peptide‐based vaccines,^35^ exome‐based vaccines,^36^ whole‐cell vaccines,^37^, ^38^ dendritic cell vaccines,^39^, ^40^, ^41^, ^42^ Toll‐like receptor agonists,^43^ DNA vaccines,^44^ agonist antibodies specific for the co‐stimulatory molecules, e.g. OX‐40^28^ and 4‐1BB,^45^ and anti‐PD‐1‐blocking antibodies.^46^ Moreover, administration of CY before adoptive T‐cell transfer significantly improves the performance of the transferred cells.^5^

The finding that antibiotics compromised the efficacy of low‐dose CY indicated that its ability to confer anti‐tumour immune effects is also dependent on the presence of certain microbes in the gut. Findings from mouse models indicate that key players include the Gram‐positive bacteria, *Enterococcus hirae* and the Gram‐negative bacteria, *Barnesiella intestinihominis*.^47^ Although *E. hirae* was shown to promote induction of memory Th1 responses and induction of pathogenic Th17 (defined as Tbet^+^ ROR*γ*T^+^ IFN‐*γ* ^+^ IL‐17^+^) cells in CY‐treated tumour‐bearing mice, *B. intestinihominis* was associated with a systemic increase in polyfunctional CD4^+^ and CD8^+^ T cells and a tumour‐localized increase in numbers of interferon‐*γ*‐producing T cells. Significant increases in cancer‐specific T‐cell responses are associated with better control of tumour growth. Why the presence of these bacteria should alter the type and size of immune responses induced because of CY treatment is not yet known. It is interesting, however, that the efficacy of CY in tumour‐bearing mice previously pre‐treated with antibiotics, was partially restored upon oral gavage comprising either organism.^47^ These findings open exciting new clinical opportunities for combining CY treatment with pre‐conditioning/colonization of the gut with bacteria that maximize the therapeutic potential of immunomodulatory drugs.

## Cyclophosphamide and metastasis – mouse models

There are opposing reports regarding the effect of CY on cancer metastasis, with some suggesting the enhancement of malignancy after CY treatment.^48^, ^49^, ^50^, ^51^ Carmel and Brown injected cytotoxic chemotherapeutic drugs into mice 1 day before inoculation with large numbers of tumour cells to assess the effect on tumour take.^51^ CY appeared to have the most significant effect on increasing the number of lung tumour nodules compared with other commonly used drugs. Interestingly, there was a sharp increase in tumour nodules above a CY dose of 100 mg/kg, with a maximum dose of 200 mg/kg resulting in a significant increase in number, possibly as a result of an effect of CY on the lung tissue.^51^ CY treatment in mice has also been observed to cause vascular endothelial cell damage, allowing for an increase in fibrosarcoma cell adhesion and embedding in the endothelial membrane, potentially enhancing metastasis.^50^ Yamauchi *et al*.^49^ found that after CY pre‐treatment, mouse fibrosarcomas became less stable, and were able to extravasate and form metastatic nodules. More recently Park *et al*.^48^ showed that a single dose of 350 mg/kg CY in mice 7 days before intracardiac injection of luciferase‐labelled PC‐3 carcinoma cells resulted in increased metastasis in hind limb and mandible compared with saline‐treated mice, and fewer metastatic‐free mice 6 weeks after tumour challenge.^48^

In contrast, Jia and Waxman showed that low‐dose metronomic CY administration could sensitize KM12 colon carcinoma to the anti‐tumour and anti‐metastatic effects of thrombospondin‐1 and pigment epithelium‐derived factor, although CY itself did not promote tumour regression.^52^ These disparate findings demonstrate how high doses of CY appear cytotoxic, whereas metronomic CY administration can be administered for prolonged periods of time with minimal side effects to maximize the anti‐tumour response.^52^

## Cyclophosphamide for cancer immunotherapy – clinical studies

Attempts to compare mouse and human trials using CY can be made by normalizing to body surface area to calculate animal and human equivalent dose. From these calculations, the doses used in mice and humans to achieve immune‐modulation are comparable. It remains difficult, however, to extrapolate dosing regimens from mouse to humans due to differences in serum half‐life (the serum half‐life of CY is < 17 min in mice and 6·5 hr in humans).^53^, ^54^ Similarly, comparing different human studies is also challenging because of the consequences of different routes of administration and dosing regimens. Nevertheless, patterns are emerging regarding the impact of low‐dose CY on the human immune system.

Some of the earliest reports to measure CY activity on Treg cells in humans involved irradiated cancer cell vaccines in patients with metastatic melanoma. A dose of 300 mg/m^2^ (approximately 8·11 or 101·38 mg/kg in a mouse) CY was found most effective at decreasing the putative suppressor T‐cell count.^55^, ^56^ However, incomplete understanding of the phenotype and function of Treg cells limited these studies at this time.

Several groups subsequently used a daily oral low‐dose metronomic regimen of CY, originally as a salvage therapy in end‐stage cancer patients, aimed at inhibiting tumour angiogenesis.^57^, ^58^ An important study by Ghiringhelli *et al*. determined that using CY in this manner actually augments anti‐tumour immunity in such patients through selective effects on CD4^+^ CD25^+^ Treg cells, allowing for greater tumour control.^59^ A number of studies have since used similar metronomic dosing regimens to limit Treg cell function to unmask tumour‐specific T‐cell responses.^60^, ^61^ The dose of CY appears crucial to its immune‐potentiating effects as demonstrated in a clinical trial involving 28 patients with metastatic breast cancer: HER2‐specific antibody responses were enhanced following vaccination with a granulocyte–macrophage colony‐stimulating factor‐secreting breast tumour vaccine and CY administered at a dose < 200 mg/m^2^ but higher dosages of CY suppressed these responses.^62^ With oral administration, 100 mg given twice‐a‐day depleted all lymphocyte sub‐populations, whereas 50 mg twice‐a‐day was selective for Treg cells.^59^ These studies serve to highlight the narrow therapeutic window of CY on anti‐tumour immune responses, corresponding with data collected using mouse models.

Several clinical trials using CY have been conducted. Greten *et al*.^63^ assessed the effect of an intravenously administered dose of 300 mg/m^2^ CY in conjunction with a telomerase peptide vaccine in hepatocellular carcinoma patients, but no definitive conclusions as to its efficacy could be drawn. However, an identical dose before treatment with a renal cell cancer vaccine (IMA901) had a significant and prolonged effect on Treg cell proliferation, reducing their overall number.^64^ In addition, a trend for prolonged survival was seen in CY‐treated patients, being one of the first randomized trials to demonstrate a beneficial effect of single‐dose CY at improving the clinical efficacy of a vaccine. A beneficial effect was not observed, however, in a phase III trial, although the results of this are confounded by addition of sunitinib as part of the drug combination, which has other, possibly conflicting, immune‐modulating effects.^65^

Some groups have also failed to observe sufficient depletion of Treg cells using iterative dosing regimens in patients with metastatic melanoma.^66^, ^67^, ^68^ In spite of these, improved anti‐tumour immune responses were reported.^66^ A study of 300 mg/m^2^ CY in ovarian cancer found neither a quantitative reduction nor qualitative difference in CD4^+^ Foxp3^+^ Treg cell function.^69^ Despite this, patients receiving CY had enhanced vaccine‐induced anti‐tumour Th1 effector responses, so displaying differential effects on potentiating vaccine immunogenicity. It is possible that the transient nature of the effect of CY on Treg cell numbers and frequencies precludes detection of the effect while the consequential increase in anti‐tumour effector T‐cell responses may be longer‐lasting.

Again, these results contrast with another study using 300 mg/m^2^ CY in melanoma patients, which reported small, transient depletions of Treg cells.^70^ The differential effect could be due to the use of CY in patients with earlier stage cancers and hence lower tumour burdens; baseline frequencies of Treg cells in this patient group, although greater than healthy controls, was statistically lower than in patients with late‐stage melanoma,^70^ a finding also observed in colorectal cancer.^61^ A recent randomized controlled phase I/II study performed by our group administered low‐dose CY with or without a modified vaccinia Ankara‐based vaccine encoding the oncofetal antigen 5T4 (TroVax) to stage IV colorectal cancer patients,^71^, ^72^ a notoriously poorly responsive tumour to immunotherapy.^73^ Compared with no treatment control patients, Treg cell depletion was observed in 24/27 (89%) patients receiving 50 mg CY twice a day, although unlike previous reports, significant B‐cell and natural killer cell depletion was also detected. Notably, significant increases in the frequency and magnitude of 5T4‐specific interferon‐*γ* ^+^ and granzyme B^+^ T‐cell responses were observed, but not for control antigens; these were associated with a significant increase in progression‐free survival.^71^, ^72^ Unexpectedly, although TroVax also induced beneficial anti‐5T4 cellular and humoral responses to the benefit of patient progression‐free and overall survival, there was no synergistic effect of combining CY with vaccine in terms of boosting observed anti‐5T4 immunological responses or patient survival.^71^ This warrants further, larger‐scale trials of CY and vaccination strategies in colorectal cancer, possibly with different treatment timings.

**Figure 1:**
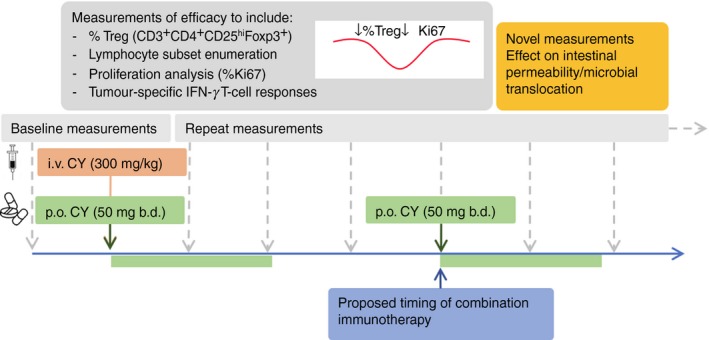
Proposal for measuring the immune‐modulating effects of cyclophosphamide (CY). Future studies of the effects of CY should incorporate baseline measurements and aim to perform analyses of immune cell subsets at many time‐points post‐administration. Such analyses should take into account numbers, proportions and function of immune cell subsets as well as cancer‐specific T‐cell responses. Measuring the impact on intestinal permeability and microbial translocation should also be considered. p.o. per oral, i.v. intravenous.

Overall, the information yielded so far regarding the impact of low‐dose CY on the human immune system is incomplete and argues for more comprehensive analyses of Treg cells in future trials. It is abundantly clear from this review that comparing studies is difficult as even when studies use the same dosage and route of administration, Treg cells are often analysed using different methods at different time‐points. This may explain why some investigators report significant effects on Treg cells whereas others do not. Indeed, in the case of the trial described above, the impact of CY on Treg cells was discovered due to the increased number of time‐points at which the cells were analysed. It is worth noting that had Treg cells been assessed just at the start and the end of the CY course, its effects would have been missed (Fig. 1).

## Conclusion

It is apparent that the dosage and method/frequency of administration are important factors determining the immune‐modulating effects of CY. Whether a given study observes depletion of Treg cells may simply depend on when measurements are made because the impact of CY on Treg cells is transient. It is probable that low‐dose CY only targets certain Treg cell sub‐populations that are actively dividing *in vivo*.^26^, ^72^

Most published data have demonstrated a beneficial effect of CY (in doses as low as 50 mg/day) in amplifying the immune response against tumours. Future trials are likely to incorporate other immune‐modifying treatments to enhance the efficacy of CY, for example in combination with novel ‘oncomicrobiotics’, or to maximize the impact of CY on potentiating anti‐tumour immune responses with co‐inhibitory receptor blockade. It remains important to define mechanisms through which these immune interventions work; CY does not always potentiate vaccine immunogenicity and studies in mice and humans imply that dosing regimens can influence the effect of CY and the efficacy of different combination approaches.^74^ In a climate where novel immunotherapies are being rapidly produced and where testing combination immunotherapies is extremely attractive, making informed choices is important. CY is an economically attractive drug and a careful reassessment of its mechanisms of action may point to a prominent role for CY in future cancer immunotherapies.

## Disclosures

The authors declare that they have no competing interests.
